# Pulmonary epithelioid inflammatory myofibroblastic sarcoma with multiple bone metastases: case report and review of literature

**DOI:** 10.1186/s13000-015-0358-1

**Published:** 2015-07-16

**Authors:** Xinge Fu, Juhong Jiang, Xiao-ying Tian, Zhi Li

**Affiliations:** Department of Pathology, The First Affiliated Hospital, Guangzhou Medical University, 151, Yanjiang Road, Guangzhou, 510120 China; School of Chinese Medicine, Hong Kong Baptist University, 7, Baptist University Road, Kowloon Tong, Hong Kong China; Department of Pathology, The First Affiliated Hospital, Sun Yat-sen University, 58, Zhongshan Road II, Guangzhou, 510080 China

**Keywords:** Epithelioid inflammatory myofibroblastic sarcoma, Inflammatory myofibroblastic tumor, Anaplastic lymphoma kinase, Differential diagnosis, Prognosis

## Abstract

Epithelioid inflammatory myofibroblastic sarcoma (EIMS) is a rare variant of inflammatory myofibroblastic tumor with distinctive morphological features and malignant clinical behavior. Only a few such cases have been described in the literature. We report here a case of unusual pulmonary EIMS with multiple bone metastases. A 21-year-old Chinese male patient presented with complaints of general fatigue and rapid weight loss, and a huge tumor arising in the left lower lobe of lung was detected by chest computed tomography. The mass of lung was totally resected. Microscopically, the tumor cells were rounded and epithelioid in shape. Myxoid stroma and inflammatory infiltration was also present. The tumor cells were immunopositive to anaplastic lymphoma kinase (ALK) in smooth cytoplasmic pattern. Fluorescence in situ hybridization (FISH) assay revealed the presence of rearrangement of ALK gene. Three months after lung surgery, there were multiple bone metastases and intraspinal mass found by positron emission tomography. The second surgical treatment was performed to remove the intraspinal lesion. The histological and immunohistochemical features of intraspinal mass were similar to those of pulmonary tumor. The diagnosis of pulmonary EIMS with multiple bone metastases was made. To the best of our knowledge, it may be the first case of an EIMS arising in lung. Awareness of EIMS in respiratory tract and its distinctive features is important for pathologists to avoid a diagnostic pitfall caused by histologic similarities to other ALK-positive tumors. ALK inhibitor is a promising treatment for this aggressive tumor regardless of its potential acquired resistance.

## Background

Inflammatory myofibroblastic tumor (IMT) is a mesenchymal neoplasm of intermediate biological potential, which frequently occur in the lung or abdominal cavity of children and young adults. Its clinical course is relatively indolent, but may recur and rarely metastasize [1]. Recently, a rare variant of IMT with distinct morphological and immunohistochemical features, epithelioid inflammatory myofibroblastic sarcoma (EIMS), has been described by Marino-Enriquez et al. in 2011 [2]. Histologically, EIMS is characterised by epithelioid morphology and a nuclear membrane or perinuclear pattern of immunostaining for anaplastic lymphoma kinase (ALK), and Ran-binding protein 2 (RANBP2)-ALK fusion in genetic examination. Unlike conventional IMT, EIMS carries poor prognosis and is associated with rapid development of local recurrence [1, 2]. To the best of our knowledge, so far only 13 cases of EIMS have been described in the literature, and only a few cases show the distant metastasis [2–4]. Herein, we present an unusual pulmonary EIMS with multiple bone metastases occurring in a young male patient. It may be the first case of an EIMS arising in the lung. Due to its rarity, the clinical and histological features of this tumor, as well as differential diagnosis are discussed.

## Case presentation

### Clinical manifestation and management

**Fig. 1:**
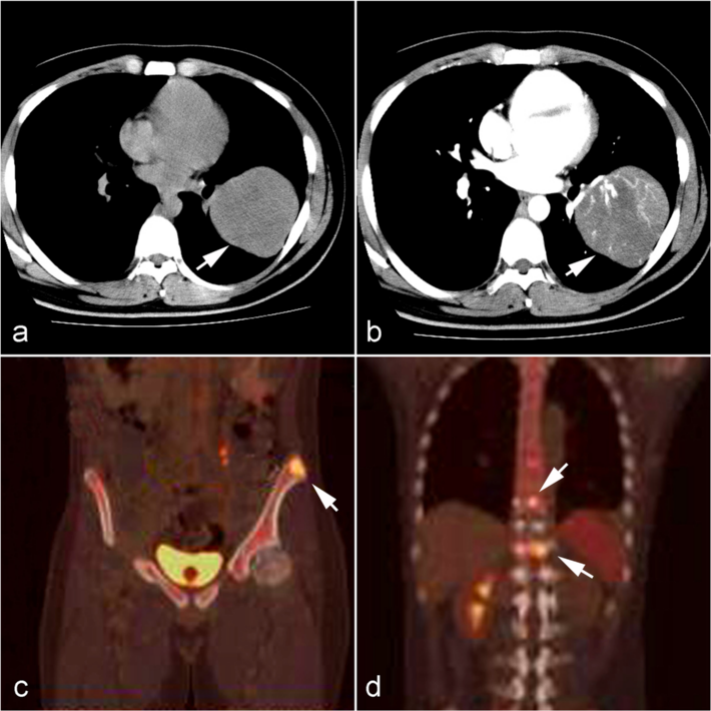
Radiological findings of lung tumor and metastatic lesions of bone. **a** Chest computed tomography (CT) revealed a huge well-circumscribed mass in the left lower lobe of lung without signs of neighboring pleura invasion (*white arrow*). **b** The pulmonary tumor showed heterogeneous enhancement. Three months after surgery, F18-fluorodeoxyglucose (FDG) positron emission tomography revealed abnormally increased FDG uptake in the pelvic bone (**c**) and vertebra mass (**d**) (*white arrows*), indicating metastatic lesions of bones

A 21-year-old non-smoking young man presented with complaints of general fatigue and rapid weight loss in the past 3 months. During this period, he was referred to a local hospital for radiological examination. Computed tomographic (CT) scans revealed a huge mass in the left lung. A fibreoptic bronchoscopy was performed in the local hospital, but biopsy examination was negative because only inflammatory mucosa was observed under the microscopy. As a result, the patient was referred to our hospital for examination and treatment. Physical examination results were normal. The laboratory results, including blood count, serum tumor markers and liver and renal function, were within the normal range. The CT images acquired at the local hospital showed a 10.0 × 8.0 cm well-circumscribed mass in the left lower lobe of lung without signs of neighboring pleura invasion (Fig. 1a and b). There was no enlarged lymph node of the pleural cavity found. Whole body F18-fluorodeoxyglucose (FDG) positron emission tomography (PET)/CT study was not preformed at that time because there was no sign of tumor distant metastasis. Since the patient was a young adult, the preoperative impression of the lesion was a mesenchymal sarcoma of lung. The patient underwent a left lobe-ectomy of lung, and the mass was gross totally resected. Postoperative recovery was uneventful without surgical complications. After diagnosis, the patient received Crizotinib, an oral anaplastic lymphoma kinase inhibitor, at a dose of 250 mg twice daily. However, the patient rapidly presented back pain and left lower limb dysfunction at the third month after pulmonary surgery. The patient was then referred to a whole body PET/CT study to search for the potentially secondary tumor. PET/CT revealed that multiple abnormally increased FDG uptake was found in pelvic bone and vertebra. An intraspinal mass at T10 was also observed (Fig. 1c and d). A second laminectomy was performed under the impression of tumor metastasis, and the majority of the intraspinal mass was resected. However, the patient developed persistent fever and cachexia. Finally, he died 4 months after the initial pulmonary surgery.

### Pathological findings

**Fig. 2:**
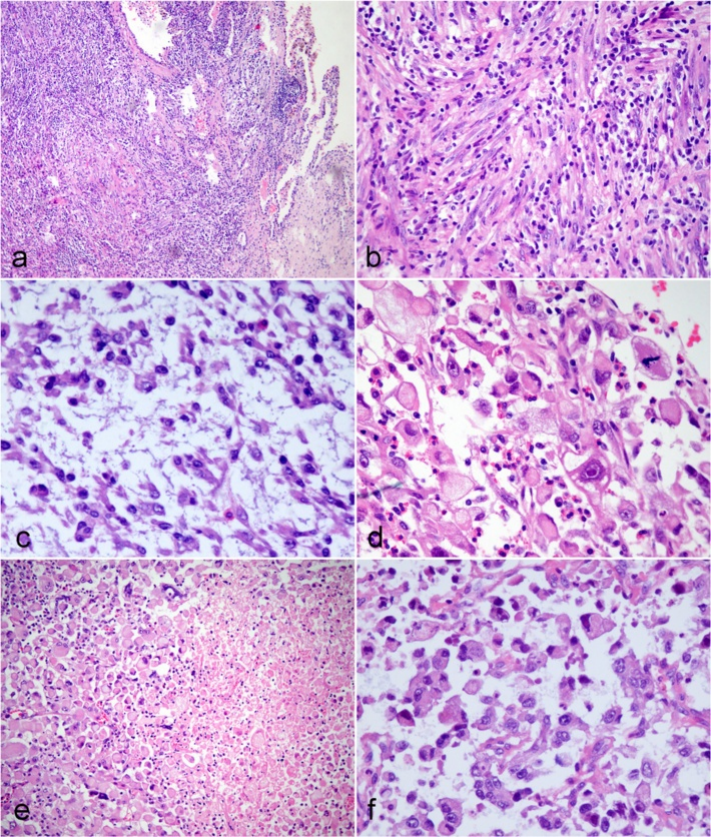
Postoperative micrographs of pulmonary and intraspinal masses. **a** Pulmonary tumor was non-encapsulated and observed to infiltrating the surrounding lung tissue. **b** In some areas of pulmonary tumor, the interlacing bundles of spindle cells were found to intermingle with plasma cells and lymphocytes. **c** The plump polygonal-like myofibroblasts with prominent nucleoli were also observed in myxoid background. **d** In other areas, the tumor was composed of diffused well-circumscribed polygonal and epithelioid tumor cells with distinct nucleoli. Nuclear atypia and mitotic figures were observed. **e** Necrosis could be found in epithelioid areas of pulmonary tumor. **f** The intraspinal tumor was composed of only polygonal and epithelioid tumor cells. **a** HE staining with original magnification × 100; **b**-**f**, HE staining with original magnification × 400)

**Fig. 3:**
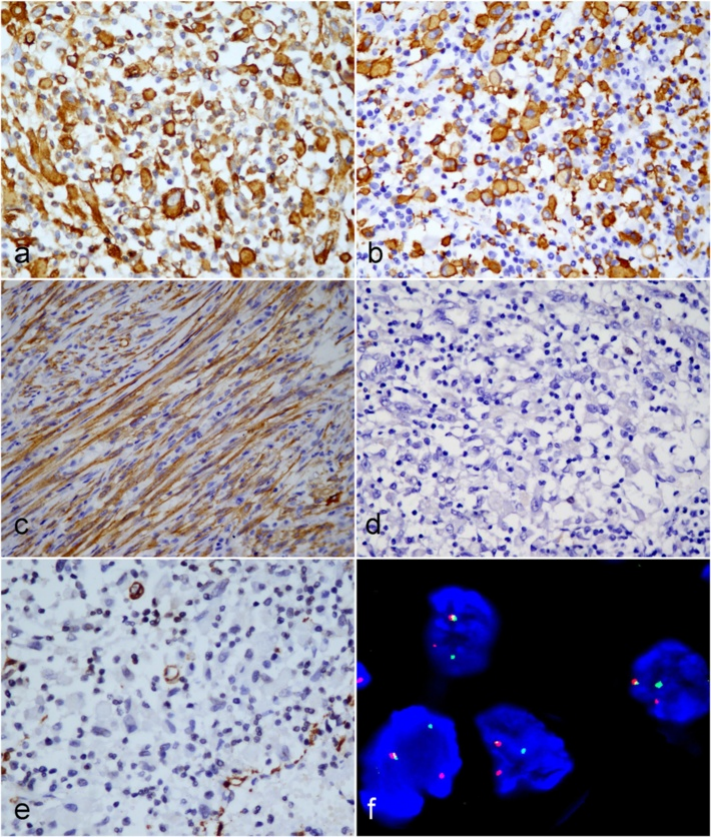
Immunohistochemical and FISH assay of tumor. The polygonal and epithelioid tumor cells were observed to be positive for Desmin diffusely (**a**) and ALK in smooth cytoplasmic pattern (**b**). **c** The spindle cells in pulmonary tumor were also found to be positive for ALK. However, the epithelioid tumor cells were negative for CD30 (**d**) and SMA (**e**) staining. **f** FISH assay with break-apart probe for ALK gene shows one intact yellow signal, one separated red and green signal per nucleus in tumor cells indicating the presence of a rearrangement of ALK gene. (**a**-**e**, immunohistochemical staining with original magnification × 400; f, FISH assay with original magnification × 400)

The surgical specimens was received and routinely fixed in 10 % neutral buffered formalin after tumor resection. Four micrometer thick sections were cut and stained with H&E. On microscopic examination, the pulmonary and intraspinal masses exhibited similar histological appearance. Both tumors were non-encapsulated and had an infiltrative margin. The pulmonary tumor was composed of two areas with distinct histological appearance. In one area, the interlacing bundles of spindle cells were found to intermingle with plasma cells and lymphocytes. The plump polygonal-like myofibroblasts with prominent nucleoli were also observed in myxoid background. The microscopical appearance of this area was consistent with the histological features of conventional IMT (Fig. 2a-c). However, in other area, the tumor was composed of diffused well-circumscribed polygonal and epithelioid tumor cells with distinct nucleoli and eosinophilic or pale cytoplasm. Nuclear atypia, mitotic figures (3/10 high power field) and focal necrosis were observed. Background of tumor in this area showed myxoid change with inflammatory cells infiltration, mainly neutrophils, eosinophils and lymphocytes. The intraspinal tumor was composed of only polygonal and epithelioid tumor cells. The spindle cell component was not present in intraspinal tumor (Fig. 2d-f).

### Immunohistochemical and FISH findings

Immunohistochemically, the tumor cells in both messes were positive for vimentin and desmin diffusely. They were negative for Pan-CK (AE1/AE3), smooth muscle actin (SMA), HHF35, myogenin, Myo D1, S100, HMB45, Melan A, synaptophysin, CD34, CD68 and CD30. In addition, the tumor cells were positive for ALK, exhibiting a cytoplasmic pattern (Fig. 3a-e). The rearrangement of the ALK gene at chromosome band 2p23 was detected by fluorescence in situ hybridization (FISH) utilizing Vysis ALK break apart probe (Vysis, Abbott Laboratories Inc., Maidenhead, UK). FISH analysis showed rearrangement of ALK present in both pulmonary and intraspinal masses (Fig. 3f).

On the basis of gross, histopathological features and immunohistochemical phenotypes, its epithelioid morphology and myxoid background, the presence of inflammatory infiltrating and ALK cytoplasmic positive signal pattern, a diagnosis of epithelioid inflammatory myofibroblastic sarcoma (EIMS) with multiple bone metastases was made.

## Discussion

EIMS is proposed as a rare variant of IMT and has firstly been designated by Marino-Enriquez et al. in 2011 because of its malignant clinical behavior [2]. Unlike conventional IMT, EIMS is characterized by plump round-to-epithelioid tumor cells embedding in abundant myxoid stroma with inflammatory infiltrate, as well as immunopositivity for ALK, and frequent RANBP2-ALK fusion gene. In 2013, the latest edition of World Health Organization (WHO) tumor classification of soft tissue (4th edition) has accepted EIMS as a variant of IMT, and identified it “seems to portend more aggressive clinical behavior”, which further indicates malignant nature of this tumor, although most of conventional IMT has relatively indolent clinical course with low rate of metastasis [1].

Since its first description by Marino-Enriquez et al. so far only 13 such cases have been described in the literature (Table 1) [2–4]. Of these 3 were children and 10 were adults with obvious male predilection, only 1 case occurred in female patient. The age range for all reported cases is from 7 months to 63 years (mean age 33y). Almost all of tumors were intra-abdominal, including the mesentery of the small bowel, omentum and peritoneum. Only 1 case arises from pleural cavity [3]. To the best of our knowledge, our case is the first case of primary pulmonary EIMS. Clinically, EIMS presents as a rapid growing intra-abdominal mass or pleural cavity nodule, it usually comes to medical attention when presence of abdominal pain, ascites or pleural effusion. Cases of reported EIMS have ranged in size from 6 to 26 cm [2–4]. In the present case, general fatigue and weight loss pain is the primary clinical manifestation instead of abdominal pain and a palpable nodular lesion.

**Table 1:** Clinicopathological features of EIMSs described in present and previous reports

No.	Authors (yr.)	Age (year)/gender	Location/size (cm)	Clinical presentation	Immuno-phenotype	Molecular change	Recurrence	Metastasis	Treatment	Outcome
1	Marino-Enriquez A (2011) [2]	59/Male	Mesentery/15.0	Abdominal pain and/or ascites	Des+, SMA+, ALK+(N)	ALK-rearrangement (FISH)	Yes	None	SE + CT	DOD 12 months after SE
2		41/Male	Omentum/26.0		Des+,CD30+, ALK+(N)	ALK-rearrangement (FISH)/RANBP2 -ALK fusion (PCR)	Yes	Liver	SE + CT + ALKi	ANED 40 months after SE
3		6/Male	Omentum/10.5		Des+, SMA-, ALK+(N)	ALK-rearrangement (FISH)	Yes	None	SE + CT	AWD 13 months after SE
4		28/Male	Mesentery/NA		Des+, SMA+, ALK+(N)	NA	NA	NA	NA	NA
5		63/Male	Mesentery/25.0		Des+,CD30+, ALK+(P)	ALK-rearrangement (FISH)	Yes	None	SE + CT	DOD 3 months after SE
6		42/Male	Intra-abdominal/NA		Des+, ALK+(N)	NA	Yes	None	SE + CT	AWD 13 months after SE
7		7 mo/Male	Peritoneum/10.0		Des+,CD30+, ALK+(N)	ALK-rearrangement (FISH)	Yes	None	SE + CT + RT	DOD 36 months after SE
8		40/Male	Peritoneum/8.0		Des-, CD30+, ALK+(N)	ALK-rearrangement (FISH)	Yes	Lung, liver, lymph node	SE + CT + RT	DOD 28 months after SE
9		31/Female	Mesentery/17.5		Des+,CD30+, ALK+(N)	ALK-rearrangement (FISH)	Yes	None	SE + CT	DOD 11 months after SE
10		6/Male	Omentum and mesentery/14.0		Des+,CD30+, ALK+(N)	ALK-rearrangement (FISH)/RANBP2 -ALK fusion (PCR)	NA	NA	SE	NA
11		39/Male	Mesentery/15.0		Des+,CD30+, ALK+(N)	ALK-rearrangement (FISH)/RANBP2 -ALK fusion (PCR)	NA	NA	SE	NA
12	Kozu Y (2014) [3]	57/Male	Pleural cavity / NA	Dyspnea and pleural effusion	Vim+, Des+, CK+, CD30-, ALK+ (C)	RANBP2-ALK fusion (PCR)	NA	NA	Biopsy + ALKi	NA
13	Kimbara S (2014) [4]	22/Male	Pelvis/6.0	Fever, fatigue, abdominal pain	Des+, SMA+, ALK+ (N)	RANBP2-ALK fusion (PCR)	Yes	Peritoneal dissemination	SE + Crizotinib	ANED 10 months after Crizotinib
14	Present case	21/Male	Lung/10.0	Fatigue and weight loss	Des+, CD30-, ALK+ (C)	ALK-rearrangement (FISH)	No	Multiple bone metastases	SE + Crizotinib	DOD 4 months after initial SE

*SE* surgical excision, *CT* chemotherapy, *RT* radiotherapy, *DOD* dead of disease, *ANED* alive, no evidence of disease, *AWD* alive with disease, *NA* data not available, *Vim* Vimentin, *Des* Desmin, *ALK* anaplastic lymphoma kinase, *ALKi* anaplastic lymphoma kinase inhibitor, *(C)* cytoplasmic pattern, *(N)* nuclear membrane

Since cases of EIMS are so rare, the diagnosis should be only made by strict histological and clinical manifestation. Based on the previously reported cases, the common histological features of EIMS are plump round-to-epithelioid cell morphology and abundant myxoid stroma with inflammatory infiltrating. Immunopositivity for ALK consistently and CD30 frequently has also been identified in most of cases [2–4]. However, these criteria can differentiate this neoplasm from epithelial-origin tumors, the same criteria could not differentiate the lesion from several mesenchymal tumors, especially from extra-nodular anaplastic large cell lymphoma (ALCL). The differential diagnosis between EIMS and ALCL can be particularly difficult, especially considering that the rare sarcomatoid variant of ALCL can show spindle cell morphology and an overlapping immunophenotype of ALK, CD30 positive staining, and SMA, EMA negative staining [5]. However, diffuse expression of Desmin in tumor cells have demonstrated in all reported cases, including our presenting case, which is not seen in ALCL. Moreover, there are three ALK staining patterns: distinctive nuclear membrane staining, granular cytoplasmic staining and smooth cytoplasmic staining, as seen in the present case. Some researchers suggest that these staining patterns are associated with RANBP2-ALK, CLTC-ALK and TPM3/4-ALK fusion partner, respectively [6]. The RANBP2-ALK fusion is considered specific to EIMS and has not been reported in ALCL. Therefore, the distinctive nuclear membrane pattern of ALK staining may be useful for differential diagnosis of these two tumors. The differential diagnosis with the solid variant of alveolar rhabdomyosarcoma, especially those examples showing ALK-positive, can also be difficult [7]. However, absence of myxoid stroma and inflammatory infiltrating, and nuclear immunoreactivity for myogenin and Myo-D1 are helpful to confirm the diagnosis of rhabdomyosarcoma.

In our case, ALK rearrangement has been confirmed by FISH assay. However, certain ALK gene fusions can not be identified unless more sensitive molecular technique, such as PCR assay or gene sequencing is performed for that detection. In fact, various partner genes have been identified to fuse with 3′kinase region of ALK gene. Nucleophosmin (NPM)-ALK fusion occurring ALCLs has been reported to be detected in approximately 50 % of IMTs [8]. Besides RANBP2, tropomyosin 3 (TPM3) and tropomyosin 4 (TPM4), clathrin heavy chain (CLTC), cysteinyl-tRNA synthetase (CARS), 5-aminoimidazole-4-carboxamide ribonucleotide formyltransferase/IMP cyclohydrolase (ATIC) and SEC31L1 have also been identified to fuse with ALK gene, and provide active promoters for the fusion gene [9–12]. Several reports have suggested that IMTs with RANBP2-ALK fusion usually exhibit an epithelioid/round cell morphology and follow a more aggressive clinical course [13, 14]. Recently, echinoderm microtubule-associated protein-like 4 (EML4)-ALK fusion and vinculin (VCL)-ALK fusion have also been identified in non-small cell lung cancer (NSCLC) [15] and renal cell carcinoma [16], respectively, which has not been described in IMT or EIMS yet. It has proposed that these tumors be collectively referred to as “ALKoma”, in which ALK genetic changes play essential roles in carcinogenesis [17].

The EIMS appears to have a poor prognosis with short survival time after surgical treatment and following chemotherapy and /or radiotherapy. Among those reported cases, 5 patients died within 3 to 36 months after the initial diagnosis, and 9 patients occurs local tumor recurrence and 3 patients had distant metastasis [2–4]. Our patient survived for only 4 months after aggressive surgical intervention and administration of crizotinib. The effectiveness of alternative treatment modalities such as radiotherapy, chemotherapy, and steroids is uncertain [18, 19]. Because EIMS frequently contain a rearranged ALK gene, ALK inhibitors are theoretically useful for treating these tumors, regardless of the site of origin. Butrynski et al. reported a case of IMT in a patient with the RANBP2-ALK fusion gene who was treated with the ALK inhibitor crizotinib after surgical tumor resection, in which 19 months have passed without evidence of recurrence [20]. Kurihara-Hosokawa [21] and Fujiya [22] recently have also presented a patient with recurred EIMS continues to be alive with disease 14 months after surgical treatment and the administration of the ALK inhibitor crizotinib. Therefore, some researchers suggest that treatment with surgery and the ALK inhibitor crizotinib may become the standard treatment for EIMS that is positive for the RANBP2-ALK fusion gene [21]. However, acquired resistance of ALK inhibitor crizotinib seems to be unavoidable. In NSCLC, ALK-positive patients develop disease progression after receiving crizotinib for 8–10 months [23]. Sasaki et al. reported IMT with RANBP2-ALK fusion gene acquired resistance to crizotinib because of secondary F1174L ALK mutation [24]. The F1174L is also found in crizotinib resistant NSCLC model [25]. The novel agents need to overcome acquired resistance to crizotinib in EIMS.

## Conclusion

In conclusion, we encountered an unusual case of pulmonary EIMS with rapidly lethal clinical course. To the best of our knowledge, it may be the first case of EIMS originating in the lung with multiple bone metastases. Due to its rarity, the diagnosis of EIMS, especially occurring in unusual sites, is difficult and should be made cautiously. Awareness of EIMS in respiratory tract and its distinctive features is important for pathologists to avoid a diagnostic pitfall caused by histologic similarities to other ALK-positive tumors. RANBP2-ALK fusion likely plays the essential role in the carcinogenesis of this tumor, and ALK inhibitor is a promising treatment for this aggressive tumor regardless of its potential acquired resistance.

## Consent

Written informed consent was obtained from the patient’s family for publication of this case report and any accompanying images. A copy of the written consent is available for review by the Editor-in-Chief of this journal.
